# Suicide and other causes of death among Chernobyl cleanup workers from Estonia, 1986 − 2020: an update

**DOI:** 10.1007/s10654-022-00957-3

**Published:** 2023-01-07

**Authors:** Kaja Rahu, Mati Rahu, Hajo Zeeb, Anssi Auvinen, Evelyn Bromet, John D. Boice

**Affiliations:** 1 Department of Registries, National Institute for Health Development, Hiiu 42, 11619 Tallinn, Estonia; 2 Formerly: Department of Epidemiology and Biostatistics, National Institute for Health Development, Tallinn, Estonia; 3 Department of Prevention and Evaluation, Leibniz Institute for Prevention Research and Epidemiology–BIPS, Bremen, Germany; 4 Faculty of Health Sciences, University of Bremen, Bremen, Germany; 5 Faculty of Social Sciences, Tampere University, Tampere, Finland; 6 Environmental Radiation Surveillance, Radiation and Nuclear Safety Authority–STUK, Tampere University, Vantaa, Finland; 7 Department of Psychiatry, Renaissance School of Medicine, Stony Brook University, Stony Brook, NY USA; 8 National Council On Radiation Protection and Measurements, Bethesda, MD USA; 9 Division of Epidemiology, School of Medicine, Vanderbilt University, Nashville, TN USA

**Keywords:** Chernobyl, Cleanup workers, Cohort, Estonia, Mortality, Suicide

## Abstract

Mortality was studied in a cohort of 4831 men from Estonia who participated in the environmental cleanup of the radioactively contaminated areas around Chernobyl in 1986–1991. Their mortality in 1986–2020 was compared with the mortality in the Estonian male population. A total of 1503 deaths were registered among the 4812 traced men. The all-cause standardized mortality ratio (SMR) was 1.04 (95% CI 0.99–1.09). All-cancer mortality was elevated (SMR 1.16, 95% CI 1.03–1.28). Radiation-related cancers were in excess (SMR 1.20, 95% CI 1.03–1.36); however, the excesses could be attributed to tobacco and alcohol consumption. For smoking-related cancers, the SMR was 1.20 (95% CI 1.06–1.35) and for alcohol-related cancers the SMR was 1.56 (95% CI 1.26–1.86). Adjusted relative risks (ARR) of all-cause mortality were increased among workers who stayed in the Chernobyl area ≥ 92 days (ARR 1.20, 95% CI 1.08–1.34), were of non-Estonian ethnicity (ARR 1.33, 95% CI 1.19–1.47) or had lower (basic or less) education (ARR 1.63, 95% CI 1.45–1.83). Suicide mortality was increased (SMR 1.31, 95% CI 1.05–1.56), most notably among men with lower education (ARR 2.24, 95% CI 1.42–3.53). Our findings provide additional evidence that unhealthy behaviors such as alcohol and smoking play an important role in shaping cancer mortality patterns among Estonian Chernobyl cleanup workers. The excess number of suicides suggests long-term psychiatric and substance use problems tied to Chernobyl-related stressors, i.e., the psychosocial impact was greater than any direct carcinogenic effect of low-dose radiation.

## Introduction

Following the accident at the Chernobyl nuclear power plant in April 1986, around 530,000 persons were involved in the cleanup activities in the radioactively contaminated territories [1]. Among them were nearly 5000 male residents of Estonia (mainly military reservists), who worked in the Chernobyl area during 1986 − 1991 [2]. We have shown previously that the general mortality in the cohort of Estonian cleanup workers did not differ from that of the male population of Estonia. An analysis of cause-specific mortality, however, revealed a significant excess of suicides [3–5].

This article extends an ongoing cohort study first assembled over 30 years ago in 1991. To our knowledge, there are no other studies evaluating cause-specific mortality, including suicides, in a well-defined cohort of Chernobyl cleanup workers. To fill this knowledge gap, particularly among those with long-term follow-up, we estimate mortality patterns using the latest available national death registration data through 2020 in Estonia.

## Data and methods

The Chernobyl cleanup worker cohort in Estonia was identified retrospectively in 1992–1994 from military and institutional records. The detailed procedures for assembling the cohort were described [2, 6, 7].

The cohort was linked to the population register to update vital status (emigration or death with the corresponding date) and to obtain ethnicity and completed educational level. Each person was followed from the date of return to Estonia (start of follow-up) until death, emigration, or 31 December 2020 (whichever came first). Cause of death information was obtained from linkage to the national mortality register. All linkages were based on the unique personal identification numbers assigned to all residents of Estonia.

Of the initial cohort of 4831 men, 19 persons (0.4%) were excluded because they could not be traced. Thus, a total of 4812 men contributing 123,420 person-years at risk of death in the period 1986–2020 were included in the analysis. Overall, 693 persons (14%) left Estonia, 91% of them were of non-Estonian ethnicity and emigrated primarily to Russia.

The overall and cause-specific death rates in the cohort were compared with those in the male population of Estonia following methods used in the previous mortality analysis [5]. The standardized mortality ratios (SMR), expressed as the ratio of observed to expected number of deaths, were calculated. The expected number of deaths in the cohort was calculated by multiplying person-years in the cohort with national male mortality rates stratified by 5-year age groups (≤ 19, 20–24, 25–29, …, 80–84, ≥ 85) and 5-year calendar periods (1986–1990, 1991–1995, …, 2011–2015, 2016–2020). The 95% confidence intervals (CI) of SMRs were computed assuming a Poisson distribution for observed deaths.

During the follow-up period, three classifications for coding causes of death were used in the mortality register: 1986–1993 the abridged Soviet classification based on ICD-9 but less detailed, 1994–1996 ICD-9, and 1997–2020 ICD-10. Cancer death risks were calculated for three groups of cancers [8]. First, radiation-related sites: salivary glands (ICD-10 C07–C08), esophagus (C15), stomach (C16), colon (C18), trachea, bronchus and lung (C33–C34), bone (C40–C41), nonmelanoma skin (C44), urinary organs (C64–C68), central nervous system (C70–C72), thyroid gland (C73) and leukemia (except chronic lymphocytic leukemia) (C91–C95, except C91.1); second, alcohol-related sites: oral cavity (C01–C08), pharynx (C09–C14), esophagus (C15), colon (C18), rectum (C19–C21), liver (C22) and larynx (C32); and third, smoking-related sites: oral cavity (C01–C08), pharynx (C09–C14), esophagus (C15), colon (C18), rectum (C19–C21), liver (C22), pancreas (C25), respiratory organs (C30–C34), urinary tract (C64–C68) and myeloid leukemia (C92). Deaths from and follow-up for cancers related to these groups could only begin in 1994.

Accordingly, mental disorders due to alcohol, degeneration of the nervous system due to alcohol, alcoholic liver disease and alcohol poisoning were grouped together to overcome the possibility of mutual misclassification of alcohol-related causes in the mortality register [9].

To determine the effect of selected characteristics on specific causes of death, the ratios of SMRs (termed relative risks, RRs) were modelled using Poisson regression with the logarithm of the expected number of cases as the offset variable [10]. In the Poisson models, the following characteristics were used: age at start of follow-up in full years (< 30, 30–39, ≥ 40 years), year of arrival in the Chernobyl area (1986, 1987–1991), duration of stay in the Chernobyl area (< 92, ≥ 92 days; median duration 92 days), time since return from the Chernobyl area (< 15, 15–24, ≥ 24 years), ethnicity (Estonian, non-Estonian), and completed educational level: higher (≥ 15 years of schooling) or secondary (11–14 years), basic or less (< 11 years). Year of arrival and duration of stay in the Chernobyl area could be interpreted as proxies for radiation exposure. Adjusted relative risks (ARR) were employed to estimate the effect of a specific characteristic, adjusting for the other characteristics.

Linkages and data analyses were done by Visual FoxPro 9.0 (Microsoft Corporation, Redmond, WA, USA) and Stata 14 (StataCorp LP, College Station, TX, USA).

## Results

Most (86.4%) cleanup workers were 20–39 years old at start of follow-up (Table 1). Nearly 61% were sent to the Chernobyl area in 1986 and half of them worked there for three months or more. Nearly half (48.9%) of the cleanup workers were of Estonian ethnicity, and one fifth had a basic or less education. By the end of 2020, 31.2% of workers (1503 men) had died and 14.4% had emigrated

**Table 1:** Characteristics of the Estonian cohort of 4812 Chernobyl cleanup workers

Characteristic	No	%
Total cleanup workers	4812	100
*Vital status on December 31, 2020*		
Living in Estonia	2616	54.4
Dead	1503	31.2
Emigrated	693	14.4
*Age at start of follow-up (full years)*		
≤ 19	80	1.7
20–29	1846	38.4
30–39	2311	48.0
40–49	541	11.2
≥ 50	34	0.7
*Year of arrival in the Chernobyl area*		
1986	2924	60.8
1987	1087	22.6
1988	564	11.7
1989–1991	109	2.3
Unknown	128	2.7
*Duration of stay in the Chernobyl area (days)*		
≤ 29	270	5.6
30–91	1997	41.5
92–149	1451	30.2
150–209	852	17.7
≥ 210	75	1.6
Unknown	167	3.5
Ethnicity		
Estonian	2354	48.9
Non-Estonian	2453	51.0
Unknown	5	0.1
Education		
Higher	396	8.2
Secondary	3059	63.6
Basic or less	963	20.0
Unknown	394	8.2

The overall mortality experience in the cohort did not differ from that of the general male population (SMR 1.04, 95% CI 0.99–1.09) (Table 2). Mortality from all cancers was elevated (SMR 1.16, 95% CI 1.03–1.28). Significant excess mortality was found for cancers of the upper aerodigestive tract and combined cancer sites related to radiation, smoking, or alcohol. After excluding the smoking- and alcohol-related cancers from the radiation-related sites, the SMR remained practically unchanged (SMR 1.20, 95% CI 0.69–1.95). No excess mortality was observed for diseases of the circulatory system (including ischemic heart disease), respiratory system or digestive system.

**Table 2:** Observed number of deaths and standardized mortality ratio (SMR) with 95% confidence interval (CI) in the Estonian cohort of 4812 Chernobyl cleanup workers by cause of death, 1986–2020

ICD-10	Cause of death	No. of deaths	SMR (95% CI)
A00–Y98	All causes of death	1503	1.04 (0.99–1.09)
A00–B99	Infectious diseases	20	0.85 (0.52–1.31)
A15–A16	Respiratory tuberculosis	18	1.15 (0.68–1.81)
C00–D48	All neoplasms	348	1.16 (1.04–1.28)
C00–C97	Cancer	343	1.16 (1.03–1.28)
C01–C15, C32	Upper aerodigestive tract	66	1.72 (1.33–2.19)
C15–C26	Digestive organs	116	1.20 (0.98–1.42)
C33–C34	Trachea, bronchus, lung	103	1.23 (0.99–1.46)
C71	Brain^a^	12	1.29 (0.67–2.25)
C73	Thyroid gland^a^	1	1.60 (0.04–8.90)
C91–C95	Leukemia	6	0.76 (0.28–1.66)
C07–C08, C15, C16, C18, C33–C34, C40–C41, C44, C64–C68, C70–C73, C91–C95 (except C91.1)	Radiation-related sites^a^	200	1.20 (1.03–1.36)
C01–C16, C18–C22, C25, C30–C34, C64–C68, C92	Smoking-related sites^a^	260	1.20 (1.06–1.35)
C01–C15, C18–C22, C32	Alcohol-related sites^a^	106	1.56 (1.26–1.86)
I00–I99	Diseases of the circulatory system	454	0.94 (0.86–1.03)
I20–I25	Ischemic heart disease	200	0.87 (0.75–0.99)
I60–I69	Cerebrovascular disease	82	1.13 (0.90–1.41)
J00–J99	Diseases of the respiratory system	67	1.14 (0.88–1.44)
K00–K93	Diseases of the digestive system	93	1.07 (0.87–1.31)
D50–H95, L00–R98	Other nonviolent causes	83	0.92 (0.73–1.14)
V01–Y98	External causes of death	408	1.09 (0.99–1.20)
V01–V99	Transport accidents	52	1.13 (0.84–1.48)
X31	Excessive cold^a^	31	1.37 (0.93–1.95)
X60–X84	Suicide	101	1.31 (1.05–1.56)
X85–Y09, Y35, Y36	Homicide	29	0.75 (0.50–1.07)
Y10–Y34	Undetermined injury	22	1.06 (0.66–1.61)
F10, G31.2, K70, X45	Selected alcohol-related causes of death (except alcohol-related cancer sites)	140	1.17 (0.98–1.37)
R99	Unknown causes	30	1.00 (0.68–1.43)

^a^Follow-up 1994–2020

There were 101 suicides in the cohort, indicating a statistically significant 31% relative excess mortality compared with the general population (SMR 1.31, 95% CI 1.05–1.56). Deaths attributed to excessive cold were elevated but not statistically significant (SMR 1.37, 95% 0.93–1.95).

The overall mortality was higher among those who worked in the Chernobyl area ≥ 92 days, were non-Estonians, or had lower (basic or less) education (Table 3). Increased all-cancer mortality was observed in the cleanup workers of non-Estonian ethnicity, or with lower education. An elevated mortality from circulatory diseases and external causes was associated with longer stay in the area, non-Estonian ethnicity, or lower education. The relative risk for suicide did not decline by time since return, and the risk was strongest among men with lower education (ARR 2.24, 95% CI 1.42–3.53). The risk of death from selected alcohol-related causes increased by time since return from the Chernobyl area.

**Table 3:** Number of deaths, crude (RR) and adjusted (ARR) relative risk of death with 95% confidence interval (CI) in the Estonian cohort of 4289 Chernobyl cleanup workers by cause of death and selected characteristics, 1986–2020^a^

Cause of death/ Characteristic	No. of deaths in the sub-cohorts		RR (95% CI)	ARR^b^ (95% CI)
	Index^c^	Reference		
*All causes of death*				
Year of arrival in the Chernobyl area 1986 vs 1987–1991	846	605	0.89 (0.79–0.98)	0.86 (0.77–0.95)
Duration of stay in the Chernobyl area (days) ≥ 92 vs < 92	769	682	1.19 (1.07–1.32)	1.20 (1.08–1.34)
Time since return from the Chernobyl area (years) 15–24 vs < 15	508	494	1.06 (0.94–1.20)	1.08 (0.96–1.23)
Time since return from the Chernobyl area (years) ≥ 25 vs < 15	449	494	0.94 (0.83–1.07)	0.98 (0.86–1.11)
Ethnicity Non-Estonian vs Estonian	715	736	1.25 (1.13–1.39)	1.33 (1.19–1.47)
Education Basic or less vs higher or secondary	481	970	1.52 (1.36–1.69)	1.63 (1.45–1.83)
*Cancer*				
Year of arrival in the Chernobyl area 1986 vs 1987–1991	184	144	0.84 (0.68–1.04)	0.84 (0.67–1.06)
Duration of stay in the Chernobyl area (days) ≥ 92 vs < 92	160	168	1.05 (0.84–1.30)	1.05 (0.84–1.31)
Time since return from the Chernobyl area (years) 15–24 vs < 15	114	63	1.05 (0.77–1.43)	1.06 (0.78–1.44)
Time since return from the Chernobyl area (years) ≥ 25 vs < 15	151	63	0.92 (0.69–1.24)	0.95 (0.70–1.28)
Ethnicity Non-Estonian *vs* Estonian	163	165	1.30 (1.05–1.61)	1.38 (1.11–1.73)
Education Basic or less *vs* higher or secondary	116	212	1.49 (1.19–1.87)	1.59 (1.26–2.02)
*Diseases of the circulatory system*				
Year of arrival in the Chernobyl area 1986 vs 1987–1991	262	182	0.94 (0.78–1.14)	0.90 (0.74–1.09)
Duration of stay in the Chernobyl area (days) ≥ 92 vs < 92	235	209	1.23 (1.03–1.49)	1.23 (1.02–1.48)
Time since return from the Chernobyl area (years) 15–24 vs < 15	157	128	0.92 (0.73–1.16)	0.93 (0.74–1.18)
Time since return from the Chernobyl area (years) ≥ 25 *vs* < 15	159	128	0.85 (0.68–1.08)	0.88 (0.69–1.11)
Ethnicity Non-Estonian *vs* Estonian	219	225	1.27 (1.05–1.53)	1.32 (1.09–1.60)
Education Basic or less *vs* higher or secondary	151	293	1.40 (1.15–1.70)	1.52 (1.24–1.87)
*External causes of death*				
Year of arrival in the Chernobyl area 1986 vs 1987–1991	225	161	0.82 (0.67–1.00)	0.82 (0.66–1.02)
Duration of stay in the Chernobyl area (days) ≥ 92 vs < 92	213	173	1.21 (0.99–1.48)	1.26 (1.02–1.55)
Time since return from the Chernobyl area (years) 15–24 vs < 15	125	217	1.23 (0.99–1.53)	1.28 (1.03–1.59)
Time since return from the Chernobyl area (years) ≥ 25 vs < 15	44	217	0.95 (0.69–1.32)	1.03 (0.74–1.43)
Ethnicity Non-Estonian vs Estonian	190	196	1.21 (0.99–1.48)	1.29 (1.05–1.58)
Education Basic or less vs higher or secondary	124	262	1.76 (1.42–2.18)	1.80 (1.44–2.26)
*Suicide*				
Year of arrival in the Chernobyl area 1986 vs 1987–1991	66	27	1.42 (0.91–2.22)	1.38 (0.86–2.22)
Duration of stay in the Chernobyl area (days) ≥ 92 vs < 92	51	42	1.19 (0.79–1.80)	1.11 (0.73–1.69)
Time since return from the Chernobyl area (years) 5–14 vs < 5	42	18	0.89 (0.52–1.55)	0.88 (0.51–1.53)
Time since return from the Chernobyl area (years) 15–24 vs < 5	23	18	0.94 (0.51–1.75)	0.93 (0.50–1.72)
Time since return from the Chernobyl area (years) ≥ 25 vs < 5	10	18	0.74 (0.34–1.61)	0.72 (0.33–1.57)
Ethnicity non-Estonian vs Estonian	37	56	0.82 (0.54–1.24)	0.91 (0.60–1.39)
Education Basic or less vs higher or secondary	33	60	2.02 (1.32–3.09)	2.23 (1.42–3.53)
*Selected alcohol-related causes of death*				
Year of arrival in the Chernobyl area 1986 vs 1987–1991	81	56	0.87 (0.62–1.23)	0.79 (0.55–1.15)
Duration of stay in the Chernobyl area (days) ≥ 92 vs < 92	77	60	1.27 (0.90–1.78)	1.34 (0.94–1.89)
Time since return from the Chernobyl area (years) 15–24 vs < 15	57	34	1.44 (0.94–2.20)	1.51 (0.98–2.32)
Time since return from the Chernobyl area (years) ≥ 25 vs < 15	46	34	1.57 (1.01–2.44)	1.69 (1.07–2.67)
Ethnicity Non-Estonian vs Estonian	65	72	1.16 (0.83–1.62)	1.20 (0.85–1.68)
Education basic or less vs higher or secondary	33	104	1.21 (0.82–1.79)	1.26 (0.83–1.90)

^a^523 subjects with unknown characteristics were excluded from the analysis

^b^Age at start of follow-up, year of arrival, duration of stay, time since return from the Chernobyl area, ethnicity, and education in the model

^c^While calculating a relative risk, the group in the place of numerator is labeled the index and of denominator–the reference

## Discussion

The cohort study of 4831 Chernobyl cleanup workers from Estonia was initially designed to estimate the effect of protracted exposure to low-dose radiation on cancer incidence, with emphasis on leukemia [7]. Subsequent research revealed that: (a) the average recorded radiation dose was relatively low, around 10 cGy, and consistent with chromosomal translocation analyses involving over a quarter of a million metaphases [11], and this low dose was less than half of the prior expectations; (b) no excess of leukemia incidence was observed [5, 12, 13], but the small number of cases and low doses were such that only 2.5-fold increases in risk could be excluded with 95% confidence [5]; c) thyroid screening of nearly 2000 cleanup workers using palpation and ultrasound found that the prevalence of thyroid nodules did not exceed a background level [14, 15]; d) cancer incidence and mortality patterns were significantly associated with ethnicity and educational level, higher among non-Estonians and among workers with lower education [5]; e) cancer sites related to smoking and alcohol were in excess [5]; f) a significantly increased 1.3–1.5-fold risk of suicide was apparent during the whole period of follow-up, from as early as 1993 [3–5]. Clinical and epidemiologic research conducted 24 years after the reactor accident found high levels of self-reported psychological distress [16] and increased risk of suicidal ideation, depressive disorders, and alcohol dependence [17].

Although the absolute number of deaths registered in our study seemed to be large, the observed mortality in the cohort was consistent (SMR 1.04) with the high premature mortality among men in Estonia [18]. Thus*,* three and a half decades after the Chernobyl accident, the most radiosensitive cancers, including leukemia and thyroid cancer, showed no detectable excess risks that might be related to low-level radiation exposure during the environmental cleanup activities. Ionizing radiation exposure during adulthood is not related to noticeable thyroid cancer risk in Japanese a-bomb survivors [19] nor in other exposed populations [14]. Although the mortality from radiation-related cancer sites was increased, the overlap of deaths that were also related to smoking and alcohol, such as lung cancer and esophageal cancer, complicates causal attribution of the excess risks.

Despite the fact that the total number of Chernobyl cleanup workers is some 530,000, few publications have addressed cause-specific mortality patterns with SMRs in a cohort setting [e.g., 20]. Our previous studies [3–5] were the first (and so far only) ones to show a significantly raised and continued risk of suicide among the Chernobyl cleanup workers.

Past radiological and nuclear accidents have repeatedly provided evidence that mental health and psychosocial consequences prevail over physical health impacts of low dose radiation exposure [21, 22]. It has been concluded that mental health issues are the major public health problem of the Chernobyl accident [23]. As discussed above, the cleanup workers from Estonia suffered from emotional distress due to their experiences manifested by symptoms of depression and anxiety, posttraumatic stress, insomnia, fatigue and somatic complaints, and alcohol problems [16]. Standardized diagnostic interviews by clinical psychologists showed an increased prevalence of depressive disorder, alcohol dependence and suicide ideation among the cleanup workers [17]. Similar results were reported for Ukrainian clean-up workers 18 years after the accident [24]. Accordingly, the excess suicide risk in our study from the earliest follow-up as a concomitant of psychiatric illness [25] can be attributed to specific disaster-related psychological stressors: sudden unexpected and forced departure from home, working in a radioactively contaminated environment in an unfamiliar country, conducting cleanup activities for no clear reason, the fear of unknown or overestimated effects of ionizing radiation, and prolonged duration of work in these rather hostile circumstances [26]. Decades after the accident, these workers continue to report traumatic memories of the Chernobyl period and attribute their health and social stressors to their experience at Chernobyl. With regard to health, as the cohort encountered aging-related diseases, a growing minority expressed frustration with the inadequate governmental support [27], likely contributing to mental health issues and elevating the risk of suicide. Increasing rates of suicide after radiation exposure have been reported throughout the lifetime of U.S. military participants at aboveground nuclear weapons tests in the 1940s and 1950s [28], indicating the need for continued evaluation of the determinants of suicide and mental disorders among the cleanup workers to target those who would benefit from counseling and preventive treatments.

A strong independent risk factor for suicide was low educational level. This may also reflect the difficulties persons with limited education had in adapting to rapidly changing circumstances after the collapse of the Soviet Union [29]. In our study, an educational gradient was found for all deaths and deaths from external causes, cancers, and diseases of the circulatory system, a pattern commonly reported in socioeconomic determinants of all-cause and cause-specific mortality, and is strongly associated with health behaviors [30–32].

Among the limitations of the study is the small size of the cohort and absence of reliable information on individual radiation doses received in the Chernobyl area, as well as the lack of extensive psychological evaluation. The small sample size limits the ability to detect associations had they existed, but nonetheless was sufficient to convincingly observe an excess of suicide and to rule out 1.7-fold increases in leukemia mortality. The recorded doses, drawn from official documents confirming participation in the cleanup operations, were biased to be high and were not useful for providing accurate estimates of exposure for individuals [33, 34]. Blood samples were collected to evaluate chromosomal translocations in circulating lymphocytes, and the loss of expression of the glycophorin A gene in erythrocytes [2, 11]. These biodosimetric evaluations confirmed that the cumulative doses received by Estonian cleanup workers were close to the threshold of detectability and the mean population dose was probably lower than the recorded dose average of 10 cGy. Blood samples were not available for the whole cohort. The low mean dose for a small population of only 4812 men indicates the very low statistical power to detect any radiation-related excesses of cancer had they occurred.

Our study benefited from the availability of national population-based registers, for which individual level data are deterministically linkable to study databases by unique personal identification numbers. The study took advantage of the continuity of a core team at the national registry committed to preserving the critical knowledge needed for conducting long-term follow-up studies. Other pluses of the study are the almost complete follow-up and the small number of deaths without a known cause. Future research will continue to monitor the long-term mortality patterns of this population, since as of December 31, 2020, just over 54% of the workers were still alive.

An unexpected obstacle took place when requesting approval for the current extension of follow-up from a local ethics committee. Formerly, use of personal health data for research purposes without a consent required permission from the Data Protection Agency (DPA), which consulted an ethics committee during the authorization procedure. According to the new Personal Data Protection Act that entered into force in Estonia on January 15, 2019, the entire research authorization procedure was delegated to the ethics committees [35] even if they had no expertise in data protection and had only a vague idea of the design and execution of longitudinal studies. In our case, the committee, referring to the General Data Protection Regulation, regarded pseudonymized data as strictly personal data [36], and, to reduce the risk of identifying dead individuals, agreed to pass individual death certification files to researchers only after anonymization. Even then, it was difficult to convince the committee that anonymized data (extracted from the causes of death register for all deaths in the male population of Estonia between 1986 and 2020) for calculating SMRs do not violate the privacy regulation. The committee also proposed to shorten the follow-up period or limit the study to lung cancer – as if reducing the amount of data processed might somehow protect privacy to an acceptable degree.

The future research on Estonian Chernobyl cleanup workers has few options other than to involve a new generation of epidemiologists. It must be hoped that these young and skilled professionals also possess or develop the patience, perseverance and wisdom necessary for overcoming the existing and emerging challenges to conduct health research. The need to attract, train and engage young people in the radiation sciences is a worldwide concern [37–39].

## Conclusions

Our findings demonstrate that 35 years after the Chernobyl nuclear power reactor accident, the overall mortality among 4831 cleanup workers from Estonia did not differ from that of the general male population. No excess cancer mortality attributable to radiation exposure was evident. The persistent risk of suicide in the cohort that was apparent as early as 1993 confirms the long-term psychosocial consequences of Chernobyl experiences among the cleanup workers.
